# Thromboangiitis Obliterans (Buerger's Disease)

**Published:** 2015-04-10

**Authors:** Nicholas R. Sinclair, Donald R. Laub

**Affiliations:** ^a^University of Vermont College of Medicine, Burlington, Vermont; ^b^University of Vermont Medical Center, Burlington, Vermont

**Keywords:** thromboangiitis obliterans, Buerger's disease, vasculitis, upper extremity, amputation

## DESCRIPTION

**Figure 1:**
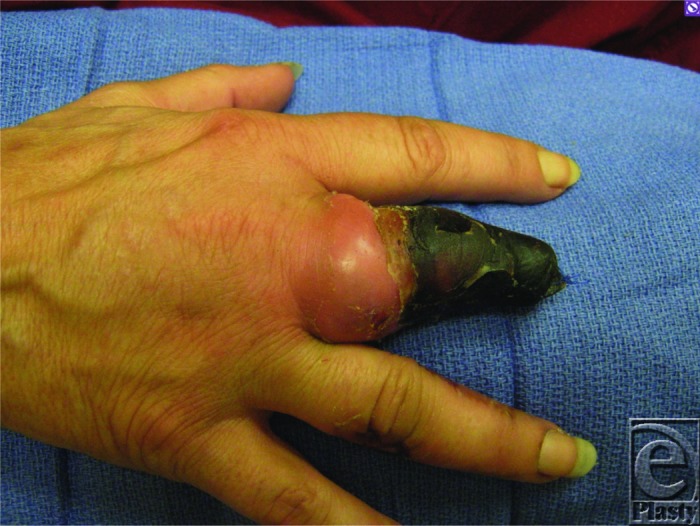
Black and gangrenous eschar tissue in the right third digit previously amputated to the middle phalanx with distal gangrene and erythema around the right middle metacarpophalangeal joint.

**Figure 2:**
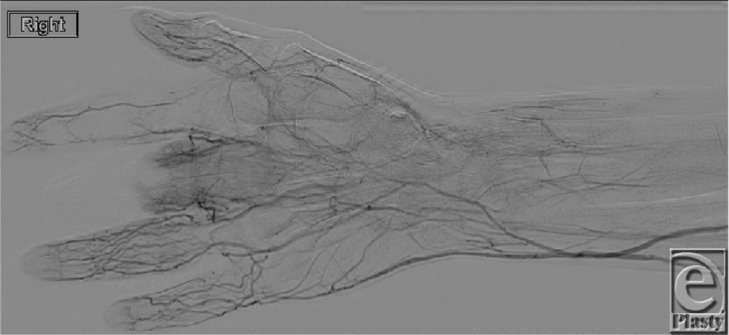
Angiography of right upper extremity showing high-grade stenosis in the distal radial artery with multiple tiny filling defects. The common and proper palmar digital arteries are heavily diseased with multiple occlusions. The princeps pollicis artery is occluded with reconstitution of the first digital artery from collaterals. Remaining digital arteries are heavily diseased with multiple occlusions. The heavily diseased third digital arteries supply the partially amputated third digit.

**Figure 3:**
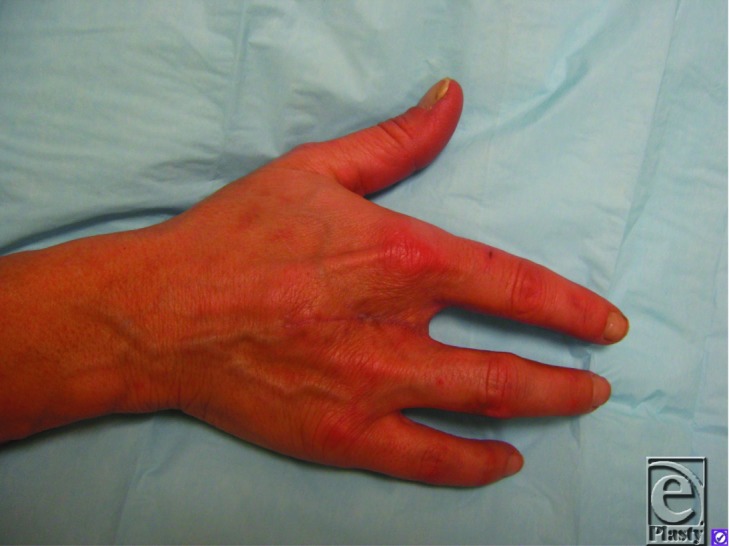
Ten weeks after ray amputation of the right third digit.

A 43-year-old white woman with a recent partial amputation at another hospital due to a nonhealing fingertip wound presented with gangrene of the residual digit and associated swelling and erythema (Fig 1). Angiography showed heavily diseased arteries of the right hand (Fig 2), and a diagnosis of thromboangiitis obliterans or Buerger's disease was made. After she ceased tobacco use, a ray amputation of the right third digit was performed (Fig 3).

## QUESTIONS

1. **What is that pathogenesis of thromboangiitis obliterans?**
2. **What is the clinical presentation of thromboangiitis obliterans?**
3. **How is thromboangiitis obliterans diagnosed?**
4. **How is thromboangiitis obliterans managed?**

## DISCUSSION

Thromboangiitis obliterans is an inflammatory disease of small- to medium-sized blood vessels of the extremities. In contrast to the other vasculitides, thromboangiitis obliterans is characterized by an inflammatory response that produces a cellular intraluminal thrombus without involvement of the vessel wall. While the disease is still poorly understood, 3 phases have been described. In the acute phase, an occlusive inflammatory thrombus comprising polymorphonuclear leukocytes, multinucleated giant cells, and micro-abscesses develops in the distal extremities. The internal elastic lamina is spared, and fibrinoid necrosis is not present. In the subacute phase, inflammatory cells are still present and the thrombus becomes increasingly organized. The chronic phase is characterized by fibrosis and an organized thrombus without inflammatory cells. Proposed causes include delayed type hypersensitivity to collagen secondary to cigarette smoking (termed “toxic angiitis”),^1^ development of antiendothelial antibodies,^2^ and hypercoagulable states (prothrombin gene mutation 20210 and anticardiolipin antibodies).^3^^,^^4^

Thromboangiitis obliterans classically develops in male smokers younger than 45 years. Two or more extremities are usually affected.^5^ Signs and symptoms are related to impaired blood flow and local ischemia. The most common symptom is pain at rest and claudication in the affected hands or feet.^5^ Other symptoms include cold insensitivity, diminished peripheral pulses, cyanosis, skin atrophy, and reduced hair growth. As the disease progresses, patients will develop ischemic ulcerations and eventually gangrene (Fig 1). In some cases, superficial thrombophlebitis can precede the onset of pain and ischemia.^5^

While definitive diagnosis is made by biopsy showing the acute-phase inflammatory cell thrombus, this is not always possible and usually not necessary. In most cases, thromboangiitis obliterans is diagnosed clinically. Common clinical diagnostic criteria include age less than 45 years, history of tobacco use, vascular testing demonstrating distal extremity ischemia, and arteriographic evidence consistent with thromboangiitis obliterans (lack of atherosclerosis, segmental occlusion, collateralization around occlusions, and lack of proximal source of thromboembolism).^5^ Serological markers of autoimmune disease and a hypercoagulability screen should be performed to rule out alternative diagnoses. As mentioned earlier, prothrombin gene mutation 20210 and anticardiolipin antibodies have been associated with thromboangiitis obliterans and do not establish the diagnosis.

The definitive treatment of thromboangiitis obliterans is tobacco cessation. Pharmacotherapy (bupropion or varenicline) and group therapy should be offered. Nicotine replacement should be avoided, as this may continue the progression of the disease.^5^ While thromboangiitis obliterans will remit with tobacco cessation, irreversible ischemic changes of tissue loss and gangrene will not. These complications are usually treated with amputation of the affected area (Fig 3). Iloprost, a prostaglandin agonist, has been shown to be more effective than lumbar sympathectomy in managing pain and healing ulcers, especially before tobacco use has been fully stopped.^6^ Recent research in therapeutic angiogenesis, including intramuscularly administered VEGF (vascular endothelial growth factor)^7^ and autologous bone marrow mononuclear cell implantation,^8^ has shown promise but are not yet utilized clinically.

Thromboangiitis obliterans should be suspected in any patient using tobacco products who presents with new-onset superficial thrombophlebitis or distal extremity pain. Initial evaluation should consist of a thorough history and physical examination. Laboratory testing should be done to rule out diabetes, autoimmune disease, or hypercoagulability as the cause. Vascular imaging should be performed to rule out atherosclerosis or embolic disease as the cause. Early diagnosis is imperative in the management of thromboangiitis obliterans, as prompt tobacco cessation will halt the progression of disease and the need for future amputations.

## References

[1] Papa M, Bass A, Adar R (1992). Autoimmune mechanisms in thromboangiitis obliterans (Buerger's disease): the role of tobacco antigen and the major histocompatibility complex. Surgery.

[2] Eichhorn J, Sima D, Lindschau C (1998). Antiendothelial cell antibodies in thromboangiitis obliterans. Am J Med Sci.

[3] Avcu F, Akar E, Demirkilic U, Yilmaz E, Akar N, Yalcin A (2000). The role of prothrombotic mutations in patients with Buerger's disease. Thromb Res.

[4] Maslowski L, McBane R, Alexewicz P, Wysokinski WE (2002). Antiphospholipid antibodies in thromboangiitis obliterans. Vasc Med.

[5] Olin JW (2000). Thromboangiitis obliterans (Buerger's disease). N Engl J Med.

[6] Bozkurt AK, Cengiz K, Arslan C (2013). A stable prostacyclin analogue (iloprost) in the treatment of Buerger's disease: a prospective analysis of 150 patients. Ann Thorac Cardiovasc Surg.

[7] Isner JM, Baumgartner I, Rauh G (1998). Treatment of thromboangiitis obliterans (Buerger's disease) by intramuscular gene transfer of vascular endothelial growth factor: preliminary clinical results. J Vasc Surg.

[8] Matoba S, Tatsumi T, Murohara T (2008). Long-term clinical outcome after intramuscular implantation of bone marrow mononuclear cells (Therapeutic Angiogenesis by Cell Transplantation [TACT] trial) in patients with chronic limb ischemia. Am Heart J.

